# Identification and characterization of Piwi-interacting RNAs in human placentas of preeclampsia

**DOI:** 10.1038/s41598-021-95307-w

**Published:** 2021-08-03

**Authors:** Jie He, Miaomiao Chen, Jiacheng Xu, Jie Fang, Zheng Liu, Hongbo Qi

**Affiliations:** 1 Department of Obstetrics, The First Affiliated Hospital of Chongqing Medical University, Chongqing, 400016 China; 2 Maternal and Child Health Hospital of Hubei Province, No. 745 Wuluo Road, Hongshan District, Wuhan City, 430070 Hubei Province China; 3 Chongqing Key Laboratory of Maternal and Fetal Medicine, Chongqing Medical University, Chongqing, 400016 China; 4 China-Canada-New Zealand Joint International Research Laboratory of Reproduction and Development of Chinese Ministry of Education, Chongqing Medical University, Chongqing, 400016 China

**Keywords:** RNA, Biomarkers, Diseases, Pathogenesis

## Abstract

Preeclampsia is a common disease of pregnancy that poses a serious threat to the safety of pregnant women and the fetus; however, the etiology of preeclampsia is inconclusive. Piwi-interacting RNAs (piRNAs) are novel non-coding RNAs that are present at high levels in germ cells and are associated with spermatogenesis. Emerging evidence demonstrated that piRNA is expressed in a variety of human tissues and is closely associated with tumorigenesis. However, changes in the piRNA expression profile in the placenta have not been investigated. In this study, we used small RNA sequencing to evaluate the differences in piRNA expression profiles between preeclampsia and control patients and potential functions. Differential expression analysis found 41 up-regulated and 36 down-regulated piRNAs in preeclamptic samples. In addition, the functional enrichment analysis of piRNAs target genes indicated that they were related to the extracellular matrix (ECM) formation and tissue-specific. Finally, we examined the expression pattern of the PIWL family proteins in the placenta, and PIWL3 and PIWIL4 were the primary subtypes in the human placenta. In summary, this study first summarized the changes in the expression pattern of piRNA in preeclampsia and provided new clues for the regulatory role of piRNA in the human placenta.

## Introduction

Preeclampsia refers to a syndrome that occurs in pregnant women after 20 weeks of gestation characterized by new onset of hypertension and proteinuria^1^. Preeclampsia affects 3% to 5% of all pregnant women and is one of the important causes of acute and chronic diseases and deaths in pregnant women and infants^1,2^. Preeclampsia poses a serious threat to the lives of pregnant women worldwide and imposes a heavy social and economic burden, especially in low-income areas and developing countries^2^. Although preeclampsia is such an important disease in obstetrics, the pathogenesis and pathophysiology of this syndrome are still controversial in academia. At present, it is widely believed that the disorder of placenta formation in the first trimester is central to the pathogenesis of this syndrome^3^. Similar to the mechanism of metastasis of malignant cells, early placental development requires proper trophoblast cell invasion of the uterus. Multiple pathogenic factors such as oxidative stress, inflammatory response, and immune intolerance are reported to be involved in the process of placental invasion. And how these factors affect placenta invasion and further lead to the occurrence of preeclampsia is an important point of the current basic research. In recent years, benefit from the development of biotechnology, genes, and their products including proteins and non-coding RNAs are found dysregulated in the preeclamptic placenta^4–6^.

piRNAs are a class of small non-coding RNAs that interacting with PIWI protein, which was first discovered in the asymmetric division of germline stem cells in Drosophila^7^. Structurally, piRNAs constitute 26–41 nucleotides and are characterized by base bias toward 5’ first uridine. They are usually located in chromatin in form of a cluster which consists of about 40–4000 piRNAs with 20–100 kb length^8^. piRNA can be derived from multiple regions on the DNA sequence, such as transposons, protein-coding regions, and specific intergenic regions^9^. piRNA forms a complex with PIWI protein and participates in the regulation of target sequences^8^. This complex can regulate the expression of other genes through a transcriptional way or a post-transcriptional way^10–12^. Specifically, piRNA directly targets the transcripts of other genes to cause their degradation or controls the expression of other genes by regulating the activity of transposons^13–15^.

At present, in addition to the gonads, piRNA has been proven to be expressed in human somatic cells^16,17^. And it has gradually become the focus of current research due to its importance in many human somatic cells and their function. Studies have shown that the occurrence and development of tumors are also closely related to the expression of piRNA^18,19^. Supported by increasingly mature sequencing technologies, the important role of piRNA in tumor pathogenesis is gradually revealed^20,21^. The mechanism of piRNA in pro-cancer and anti-cancer properties is elaborated in the existing study^20^. Recently, a study has shown that PIWIL1 protein binding to piRNA can control metastasis of pancreatic ductal adenocarcinoma^22^. Sequencing technology has also played an important role in the advancement of tumor research. A recent study has shown that PIR-Hep1 is up-regulated in hepatocellular carcinoma and can regulate the invasion and migration function of liver cells through the Akt pathway, which may be an important molecule affecting the tumor formation of hepatocellular carcinoma^23^. Besides, piRNA-30473 has also been shown to enhance HK2 methylation through WTAP, an m6A mRNA methylase, to regulate tumor genesis of diffuse large B-cell lymphoma, and was significantly associated with the prognosis of this cancer^24^. So far, there have been many studies on the association between piRNA and tumor genesis, metastasis and drug resistance, and more and more tumor prognostic prediction methods and treatment methods designed by this mechanism have been developed^25–28^.

Therefore, considering the gaps in the role of piRNA in preeclampsia studies and the similarity between the placental invasion of the uterus and tumor metastasis, we wonder whether piRNA was also involved in the pathogenesis of preeclampsia and which piRNA might be involved in the occurrence of this disease.

## Results

### Sequence signature and genome location of piRNAs in human placenta

**Figure 1:**
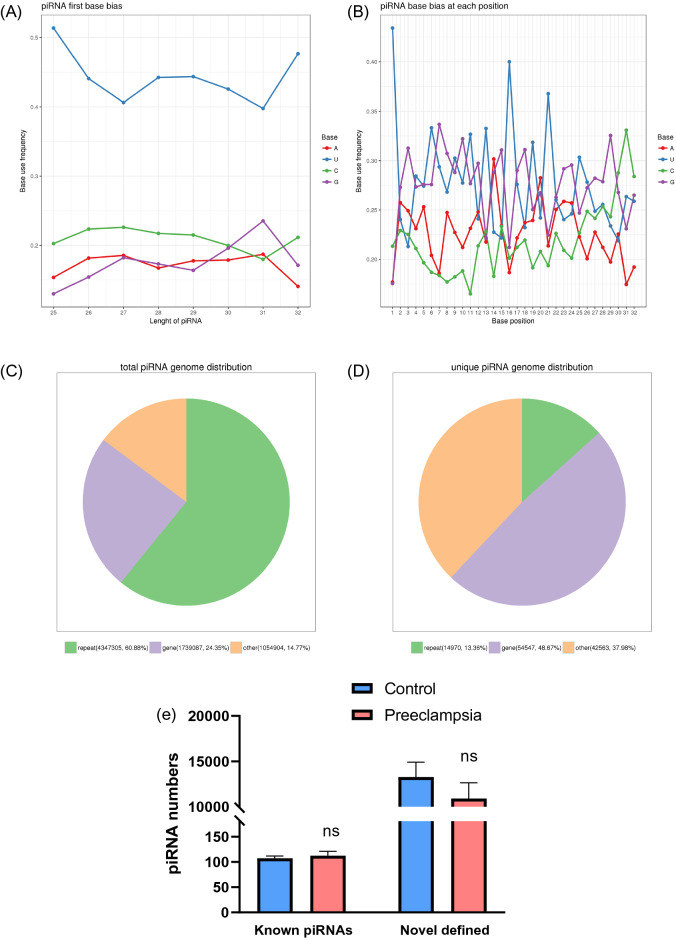
Nucleic base bias distribution and genome location of piRNAs. (**A**) Visual diagram of preference of the first base of piRNAs with different lengths. (**B**) Visual diagram of base bias at each site of the piRNA sequence. (**C**) Pie graph of total piRNAs location in the genome. (**D**) Pie graph of unique piRNAs location in the genome. (**E**) Numbers of putative piRNAs in both preeclampsia and control group.

We carried out piRNA-seq in 3 preeclamptic placentas and 3 control placentas with matched gestational age (Supplementary Table 1). In our data, sequencing fragments showed obvious main peaks at 25-32nt, a characteristic length of piRNAs. Meanwhile, we found that our putative piRNAs bear obvious uridine nucleic bias at the first 5’ nucleotide, but no bias to adenine at position 10(10A bias) (Fig. 1A,B). The obtained piRNA sequences were compared with the sequences recorded in the database, and total piRNAs were divided into known piRNAs and novel-defined piRNAs (unique piRNAs). The locations of putative piRNAs were analyzed by base alignment to the human genome. Most of the total piRNAs are located in the repeat region (Fig. 1C). About a quarter of the total piRNAs were generated from the gene region of the human genome, and the percentage was even higher in unique piRNAs, at about 50% (Fig. 1D). There was no significant difference in the numbers of piRNAs identified between the two groups (Fig. 1E).

### Dysregulated piRNAs in preeclampsia

**Figure 2:**
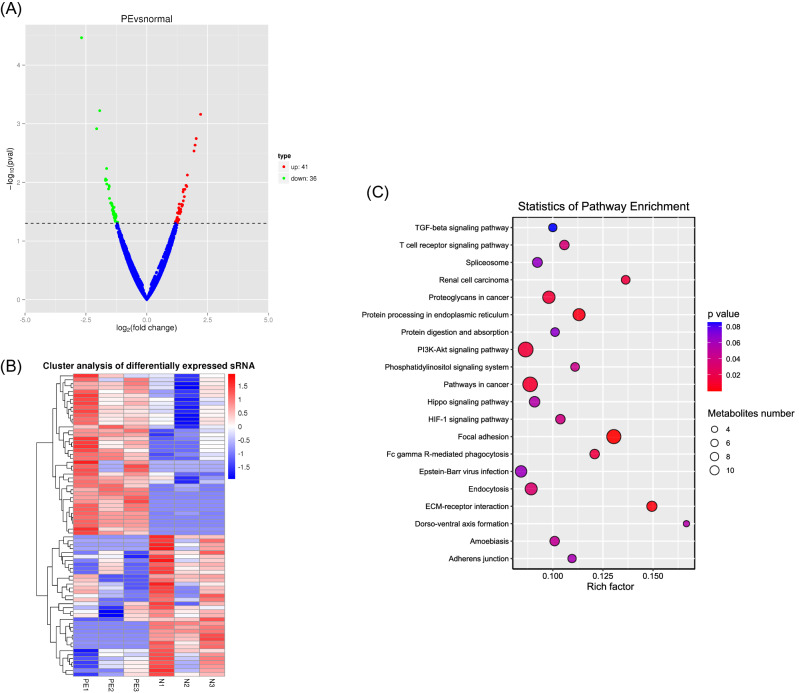
Identification of differently expressed piRNAs between preeclamptic and control placenta and pathway analysis. (**A**) Volcano plot of differentially expressed piRNAs in two groups (P-value < 0.05, log2(Fold Change) > 1). (**B**) Heatmap of the dysregulated piRNAs in two groups. (**C**) KEGG analysis of total piRNA target genes.

To study if there was a difference in piRNA expression between the two groups, we performed differential expression analysis between preeclampsia and control placentas. 77 significantly dysregulated piRNAs were found, 41 of which were up-regulated and 36 were down-regulated (Fig. 2A). The expression profile of these differentially expressed piRNAs is shown in the heatmap (Fig. 2B). Among them, 7 known piRNAs were further marked which are piR-hsa-4153, piR-hsa-8488, piR-hsa-15254, piR-hsa-16926, piR-hsa-16984, piR-hsa-20364, and piR-hsa-23338 (Table 1). KEGG results of total piRNA target genes showed that they were relevant to the PI3K-Akt pathway and some cancer-associated pathways (Fig. 2C).

**Table 1:** Top known piRNAs significantly differentially expressed between the cases and controls.

Gene Symbol	Gene description	regulation	Fold change	P-value
piR-hsa-004153	Piwi-interacting RNA 4153	Down	1.9324	0.001
piR-hsa-008488	Piwi-interacting RNA 8488	UP	1.3163	0.027
piR-hsa-015254	Piwi-interacting RNA 15,254	UP	1.5040	0.013
piR-hsa-016926	Piwi-interacting RNA 16,926	UP	1.3337	0.023
piR-hsa-016984	Piwi-interacting RNA 16,984	UP	1.5645	0.013
piR-hsa-020364	Piwi-interacting RNA 20,364	UP	1.1789	0.047
piR-hsa-023338	Piwi-interacting RNA 23,338	UP	1.2801	0.046

### Distribution and functional analysis of piRNA target genes

**Figure 3:**
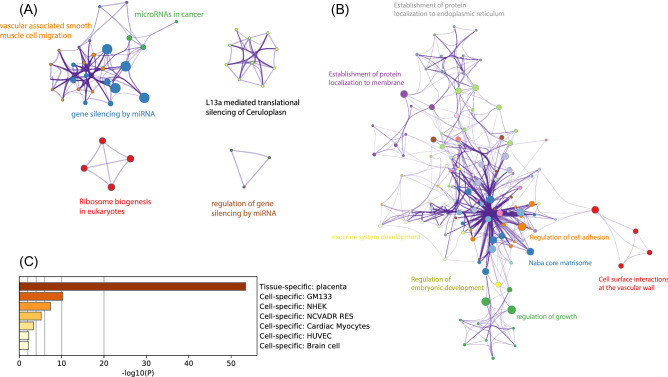
Functional analysis of piRNA target genes. (**A**) Enrichment analysis of highly expressed piRNA target genes. (**B**) Enrichment analysis of high target hit frequency genes. (**C**) Tissue-specific characteristics of high target hit frequency genes.

To better understand the roles of placenta piRNAs, we analyzed two other important sets of piRNA target genes. Firstly, we extracted the target genes of higher expressed piRNAs for enrichment analysis, and found most of them were closely related to the gene silencing mechanism of miRNAs (Fig. 3A) Next, we extracted the genes with higher piRNA hit frequency for enrichment. These target genes show better internal correlation and are related to cell basic functions such as cell growth, cell adhesion, and especially cell ECM formation such as NABA CORE MATRISOME (Fig. 3B). Interestingly, pathways associated with the development of the embryo, and exocrine system were also enriched. (Fig. 3B). It’s worth noting that most of these genes were expressed in the placenta in a tissue-specific manner (Fig. 3C).

### PIWIL family gene expression in preeclamptic and normal human placenta

**Figure 4:**
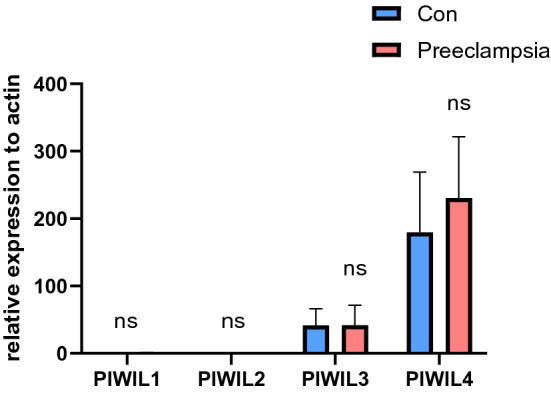
Relative mRNA expression of PIWIL family protein in 15 preeclamptic and 16 normal placentas measured by qRT-PCR (Supplementary Table 2). 2(-ΔΔCt) values were used to measure the gene expression, which was normalized according to the ACTIN expression levels. The presented values are the means ± SE, *P < 0.05, ns, no significance.

piRNAs can maintain genome structure and mRNA stability and regulate protein synthesis by binding to members of the PIWI protein family^29^. Therefore, we examined the expression of human PIWL family proteins in normal and preeclampsia placentas. It was found that the expression of PIWIL4 and PIWIL3 were higher in the human placenta, and the expression of PIWIL1 and PIWIL2 was nearly undetectable (Fig. 4). Moreover, there was no difference in the expression of either protein between preeclamptic and normal placentas.

## Discussion

Metastatic malignant cells and trophoblast cells are similar in biological behavior to a great extent^30^. Metastatic cancer cells reconstitute the ECM, invade the basement membrane, and escape immune surveillance into the bloodstream. Similarly, placenta villi, a tissue that is genetically different from the mother, invade decidua and suppress the maternal immune system. Both biological processes are accompanied by many of the same abnormal signaling pathways, such as HIF^31^. Tumor parenchymal cells proliferate rapidly and are in a relatively hypoxic environment, which makes cancer cells more likely to invade and metastasize. The same thing also happens in the placenta. Trophoblast embolization into the spiral artery in early pregnancy causes local hypoxia, which stimulates the invasion of the trophoblast and reconstruction of the uterine spiral artery^32^. It provides sufficient blood flow to the fetus. Oncology researchers are also looking for answers to the mother's immune tolerance to fetal substances during pregnancy, in the hope of providing a harmless way to treat cancer^30^. Understanding the mechanisms of tumors will help us understand the placenta. Based on this correlation we carried on our research on piRNAs in the placenta.

To investigate the gaps in the study of piRNA in the human placenta and the correlation between piRNA and preeclampsia, placental tissue was sequenced and the results were preliminarily analyzed. To our surprise, a large number of known piRNAs and unique piRNAs were identified in the human placenta. Many piRNAs were expressed differently between the two groups, but further experimental verification and functional studies are needed. Preliminary analysis reveals that piRNAs in the placenta may regulate villi invasion and be related to the expression of placenta-specific genes. Besides, they are related to the way of miRNA-like gene expression silencing. Our results suggest that piRNA may play an important role in the placenta and may be associated with the pathogenesis of preeclampsia.

As has been reported in other species, piRNAs in the human placenta we identified were also characterized by length and base preference. The 1U bias reflects the specificity of endonuclease, while the 10A bias is a character of biogenesis through the ping-pong pathway^33^. The lack of 10A preference may suggest a different biogenesis pathway in humans from Drosophila but evidence on it is rare. Moreover, most of these piRNAs were located in the genome repeat sequence region which is also a character of piRNA. The location in the repeat region or gene region in the genome suggests the function of human piRNA in transposon silencing and post-transcriptional gene silencing. From the perspective of the piRNA sequence signature and genome location, piRNAs in the human placenta were very similar to piRNAs in other species which indicated the evolutionary conservation.

A large number of piRNAs were identified in the placenta, and some were differently expressed. We wondered about the underlying functions of piRNAs in the placenta and perform KEGG analysis of total piRNA target genes and found that piRNA may be related to the cancer-associated pathway and PI3K-Akt pathway. From the point of view of cell biology, trophoblast, the main cell type of the placenta, is very similar to the cancer cell^34^. PI3k-Akt pathway regulates nutrient transports and metabolic enzymes of cancer cells supporting survival and proliferation of the malignant cell^35^. Previous studies indicated that the PI3K-Akt pathway also regulates invasiveness of trophoblast in preeclampsia^36^. piRNAs may partly participate in the pathogenesis of preeclampsia through regulating the PI3K-Akt pathway. This may provide a new way of insight into the epigenetic mechanisms of preeclampsia.

To get a deeper understanding of the meaning of piRNAs in the placenta, some important piRNA sets and their target gene sets were analyzed. Firstly, we selected the top expressed piRNAs in all samples for enrichment analysis and found these genes were closely relevant to mRNA silencing especially in a miRNA way. piRNA and miRNA derive from different RNA precursors but they both bind to Argonaute family proteins which can be classified into the AGO and PIWI clades^33^. miRNAs guide AGO proteins to form RNA-induced silencing complex to degrade their target mRNAs. Analogously, piRNAs guide PIWI proteins rather than the AGO proteins to silencing other genes in the miRNA and siRNA ways^33^. This result indicated that the major part of piRNAs in the placenta may regulate mRNA stability. Besides, the long-term action of vascular smooth muscle migration is an important pathological phenomenon of hypertension, which is also common in other vascular diseases including preeclampsia^37^. In our results, the miRNA regulation pathway is closely associated with the vascular muscle cell pathway, which suggests the underlying role of piRNAs in regulating placenta vascular muscle cells. This provides a new possibility of epigenetic regulation of vascular endothelial migration in the placenta.

The sequence length of mRNA is much longer than piRNA, so more than one piRNA may regulate common mRNA stability. Therefore, we selected the genes with higher hit frequency and analyzed their function. The core function that was enriched is Naba core matrisome, a conceptional gene collection of extracellular matrix proteins and associated factors^38^. The density and ingredients of ECM determine the outcome of cellular invasiveness and migration. Preeclampsia is characterized by the deficiency of trophoblast invasion and migration, which is a currently recognized pathological manifestation^39^. The ability of trophoblast cells to invade and migrate is finally reflected in the expression of ECM-associated factors, especially matrix metalloproteinases (MMPs) and tissue inhibitors of metalloproteinases (TIMP1 and TIMP2), which are reported decreased in preeclampsia^40–42^. Cell invasion and migration ability can be regulated by many causes including tissue inflammation and oxidative stress. Our previous studies have also shown that antioxidants can improve the symptoms of preeclampsia by regulating trophoblast invasion^43^. Our results also suggested that piRNA may be a potential regulating factor of cell invasion due to its direct mechanism of targeting ECM genes. In addition, we found that nearly a quarter of the input higher hit target genes were enriched in a term of placenta tissue specific. It demonstrated that most of the piRNAs in the placenta may target a set of placenta-specific genes. It’s an interesting phenomenon that placenta piRNA was closely related to its tissue-specific gene expression. This provides a possibility for placental-specific gene expression at the transcriptional level.

Piwi family protein is the protein part of the Piwi-piRNA complex, which forms piRNA-induced silencing complex (pi-RISC) together with piRNA to play its function of regulating the expression of other genes^33,44^. Abnormalities in the PIWI protein family have been associated with the development of a variety of cancers, such as lung cancer, renal cell cancer, and gastric cancer^45–47^. So we also wonder whether there are differentially expressed PIWI family proteins in the placenta of preeclampsia. Our results indicated that no difference was found between preeclamptic and control placenta. Besides, PIWIL1 and PIWIL2 seemed not to be expressed in the human placenta. This may suggest that piRNA in the human placenta performs its function by interacting with PIWIL3 and PIWIL4.

Although we identified the dysregulated piRNAs in preeclampsia and discussed potential functions of piRNAs in the placenta, there are still several shortages in our study. So far, some pieces of evidence demonstrate that patients with preeclampsia can be divided into two groups of early-onset and late-onset, which originate from different mechanisms, but our results lack an answer to this part. In addition, our study was primarily based on the sequencing at the gene transcription level and lack evidence for the direct interaction between piRNAs and their target mRNAs. Our next plan is to further study the specific roles of piRNA and PIWIL proteins in the human placenta and their possible effects on the pathogenesis of preeclampsia.

To sum up, in the light of our understanding, we are the first to search for piRNAs by a high-throughput approach in human placenta tissue. We detected the expression of piRNA in the human placenta and found the differentially expressed piRNAs in preeclampsia and normal human placenta by a high-throughput method. Then, we described the possible functions of these piRNA target genes and concluded that piRNAs in the placenta may regulate the ECM formation and remodeling through a miRNA-like post-transcriptional gene silencing way. Finally, we detected the expression of the PIWI protein family in the placenta and did some basic work for the further study of piRNA in the preeclampsia placenta. Our results have broadened the understanding of the active site of piRNA in the human body, increased our further understanding of preeclampsia pathogenesis, and also done some basic work for the role of piRNA in the pathogenesis of preeclampsia.

## Methods

### Patients and inclusion criteria

In our study, all placenta samples were collected from the Department of Obstetrics at the First Affiliated Hospital of Chongqing Medical University from September 2019 to September 2020. The control group was matched according to age, sex, date of blood collection, and date of delivery, and then randomly selected preeclampsia group. The diagnostic criteria for preeclampsia are as follows: new-onset hypertension, systolic blood pressure greater than 140 mm Hg or diastolic blood pressure greater than 90 mm Hg, or accompanied by significant proteinuria or multiple organ problems after 20 weeks of gestation (such as pulmonary edema, persistent upper abdominal or right upper quadrant pain with abnormal liver enzymes, thrombocytopenia, progressive renal insufficiency, new-onset cerebral or visual impairment). Exclusion criteria included the diagnosis of kidney disease, liver disease, endocrine disorders, autoimmune diseases, previous history of cervical surgery, history of pregnancy loss, known fetal abnormalities or abnormal karyotypes, and obstetric intervention at the time of recruitment. The project was reviewed and approved by the Chongqing Medical University Ethics Committee.

### Informed consent

Informed consent was obtained from all individual participants included in the study.

### Sample collection and processing

Preeclamptic and normal placental tissues were taken from the region near the position of umbilical cord insertion and were rapidly collected within 1 h after a cesarean birth. Placenta tissues at the basal plate from both healthy pregnant woman and preeclampsia patients were collected. These tissue samples were cut into small pieces using sterile scissors and washed in 0.9% cold saline, stored in RNAlater reagent (Thermo Fisher Scientific, USA) by the manufactural method at − 80 °C, and used for RNA extraction.

### RNA isolation, library preparation, and sequencing

For RNA extraction step, we used 1% agarose gels to detect the presence of RNA contamination and degradation, and NanoPhotometer spectrophotometer (IMPLEN, CA, USA) to detect the RNA purity. RNA concentration was analyzed in Qubit2.0 Fluorometer (Life Technologies, CA, USA). RNA integrity should be measuring with RNA Nano 6000 Assay Kit of the Agilent Bioanalyzer 2100 system (Agilent Technologies, CA, USA). Samples that passed the above tests can be used in follow-up steps. For libraries preparation, 3 ug total RNA was taken from each sample to prepare small RNA library. Next, we used NEBNext Multiplex Small RNA Library Prep Set for Illumina to produce sequencing libraries. (NEB, USA.) Meanwhile, index codes were added to attribute sequences of each sample. PCR was performed with LongAmp Taq 2X Master Mix, SR Primer for Illumina, and index (X) primer. The products were further separated in 8% polyacrylamide gel. (100 V, 80 min) DNA fragments of 140–160 bp in length (the length of small noncoding RNA plus the 3’ and 5’ adaptors) were extracted and dissolved in an 8 μL elution buffer. We assessed the quality of libraries on the DNA High Sensitivity Chips of Agilent Bioanalyzer 2100 system. At last, the library preparations were sequenced on Illumina Hiseq 2500/2000 platform and 50 bp single-end reads were generated.

### RNA-seq and data analysis

Raw data (raw reads) of fastq format were firstly processed through custom Perl and python scripts. In this step, clean data (clean reads) were obtained by removing reads containing ploy-N, with 5’ adapter contaminants, without 3’ adapter or the insert tag, containing ploy A or T or G or C and low-quality reads from raw data. At the same time, Q20, Q30, and GC-content of the raw data were calculated. Then, chose a certain range of length from clean reads to do all the downstream analyses. The small RNA tags were mapped to the reference sequence by Bowtie without mismatch to analyze their expression and distribution on the reference. Mapped small RNA tags were used to looking for known piRNA. piRNABank (http://piRNAbank.ibab.ac.in) was used as a reference. To remove tags originating from protein-coding genes, repeat sequences, miRNA, rRNA, tRNA, snRNA, and snoRNA, small RNA tags were mapped to RepeatMasker, Rfam database, or those types of data from the specified species itself (Supplementary Table 3). Unmapped reads were analyzed to predict the unique piRNA via piRNA predictor^48^. Custom scripts were used to obtain the piRNA counts as well as base bias on the first position of identified piRNA with certain length and on each position of all identified piRNA respectively. The total rRNA proportion has been used as a marker as a sample quality indicator. Usually, it should be less than 60% in plant samples and 40% in animal samples as high quality. piRNA expression levels were estimated by TPM (transcript per million) through the following criteria^49^: Normalization formula: Normalized expression = mapped read count/Total reads*1,000,000.

### RNA extraction and real-time quantitative PCR

Total RNA was extracted from placental tissue with TRIzol reagent (Invitrogen, USA), and the specific method was operated according to the instructions. Nanodrop (Thermo Fisher Scientific, Paisley, UK) was used to detect the purity and quantity of total RNA, and the total RNA was stored at − 80 °C. The RNA was extracted using a reverse transcription kit (MedChemExpress, USA) to obtain cDNA according to the manufacturer's instructions. The obtained cDNA and primer pairs (TsingKe, China) designed for the target gene were used and the qPCR SYBR Green Master (MedChemExpress, USA) was used for real-time quantitative PCR in the CFX96 Touch real-time PCR detection system (Bio-Rad, USA). ACTIN was selected as the reference gene for expression analysis. Then 2(-delta delta Ct) method was used to analyze Ct values. Primer sequences are shown in Table 2.

**Table 2:** Primers used for qRT-PCR.

Primer name	Orientation	Primer sequence
PIWIL1	Forward	ACGAAGTGCCACAGTTTTTGG
	Reverse	AGTCTTCCTCCAGACTGAGC
PIWIL2	Forward	GCCTGGGTTGAACTAAAGGA
	Reverse	CCATGATGATGCAAACAACC
PIWIL3	Forward	TCAGATGGCAGCAAAATCAC
	Reverse	ACGTTGTGTACCCGTTAGGC
PIWIL4	Forward	ATGGCACCGAGATCACCTAT
	Reverse	GCTGAGCCTCACTGTTGTCA
ACTIN	Forward	TGGCACCCAGCACAATGAA
	Reverse	CTAAGTCATAGTCCGCCTAGAAGCA

### Bioinformatics analysis

Differential expression analysis of two conditions/groups was performed using the DESeq R package (1.8.3). The P-values were adjusted using the Benjamini & Hochberg method. A corrected P-value of 0.05 was set as the threshold for significantly differential expression by default. Gene Ontology (GO) enrichment analysis was used on the target gene candidates of piRNAs (“target gene candidates” in the following) via Metascape (http://metascape.org)^50^. KEGG^51^ is a database resource for understanding high-level functions and utilities of the biological system, such as the cell, the organism, and the ecosystem, from molecular-level information, especially large-scale molecular datasets generated by genome sequencing and other high-throughput experimental technologies (http://www.genome.jp/kegg/). R software and ggplot2 package were used for visualization of graphics^52,53^. We used KOBAS software to test the statistical enrichment of the target gene candidates in KEGG pathways.

### Ethics declarations

The study was conducted according to the guidelines of the Declaration of Helsinki, and approved by the Ethics Committee of the First Affiliated Hospital of Chongqing Medical University (protocol code 2019-136).

## Supplementary Information

Supplementary Table 1.

Supplementary Table 2.

Supplementary Table 3.

## Data Availability

The data used to support the findings of this study are available from the corresponding author upon request.
