# Author Correction: Identification and characterization of Piwi-interacting RNAs in human placentas of preeclampsia

**DOI:** 10.1038/s41598-021-98274-4

**Published:** 2021-09-14

**Authors:** Jie He, Miaomiao Chen, Jiacheng Xu, Jie Fang, Zheng Liu, Hongbo Qi

**Affiliations:** 1 Department of Obstetrics, The First Affiliated Hospital of Chongqing Medical University, Chongqing, 400016 China; 2 Maternal and Child Health Hospital of Hubei Province, No. 745 Wuluo Road, Hongshan District, Wuhan City, 430070 Hubei Province China; 3 Chongqing Key Laboratory of Maternal and Fetal Medicine, Chongqing Medical University, Chongqing, 400016 China; 4 China-Canada-New Zealand Joint International Research Laboratory of Reproduction and Development of Chinese Ministry of Education, Chongqing Medical University, Chongqing, 400016 China

Correction to: *Scientific Reports* 10.1038/s41598-021-95307-w, published online 03 August 2021

In the original version of this Article, Affiliation 2, 3 and 4 were not listed in the correct order. The correct affiliations are listed below:

Affiliation 2:

Maternal and Child Health Hospital of Hubei Province, No. 745 Wuluo Road, Hongshan District, Wuhan City, 430070, Hubei Province, China

Affiliation 3:

Chongqing Key Laboratory of Maternal and Fetal Medicine, Chongqing Medical University, Chongqing, 400016, China

Affiliation 4:

China-Canada-New Zealand Joint International Research Laboratory of Reproduction and Development of Chinese Ministry of Education, Chongqing Medical University, Chongqing, 400016, China

The original Article has been corrected.

