# Supplementary material for: Identification and characterization of Piwi-interacting RNAs in human placentas of preeclampsia
Source: Sci Rep. 2021 Aug 3;11:15766. doi: 10.1038/s41598-021-95307-w (PMC8333249; doi:10.1038/s41598-021-95307-w)
Supplement: Supplementary file 1 — Supplementary Table 1. [file 41598_2021_95307_MOESM1_ESM.docx]

| Patient | Age | BMI | Gestational week | BP | proteinuria | Delivery mode | Complication |
| --- | --- | --- | --- | --- | --- | --- | --- |
| N17 | 24 | 14.5 | 37+1 | 95/70 | - | CS | None |
| N18 | 27 | 21.7 | 38+1 | 120/80 | - | CS | None |
| N19 | 37 | 20.2 | 34+1 | 99/64 | - | CS | none |
| PE4 | 31 | 21.3 | 38+3 | 150/90 | ++ | CS | ascites |
| PE3 | 32 | 21.7 | 38+6 | 140/81 | +++ | CS | none |
| PE16 | 30 | 21.8 | 33+6 | 145/93 | +++ | CS | ascites |

Note: BP refers to blood pressure. BMI data were collected before the pregnancy began.

Supplementary Table 1. Information of patients whose tissue sample were used for sequencing
