# Supplementary material for: Identification and characterization of Piwi-interacting RNAs in human placentas of preeclampsia
Source: Sci Rep. 2021 Aug 3;11:15766. doi: 10.1038/s41598-021-95307-w (PMC8333249; doi:10.1038/s41598-021-95307-w)
Supplement: Supplementary file 2 — Supplementary Table 2. [file 41598_2021_95307_MOESM2_ESM.docx]

| serial number | Age | Gestational age | Blood pressure | proteinuria | Delivery mode |
| --- | --- | --- | --- | --- | --- |
| N1 | 30 | 39+1 | 131/86 | - | CS |
| N2 | 34 | 39+1 | 110/65 | - | CS |
| N3 | 33 | 38+6 | 116/62 | - | CS |
| N4 | 31 | 39+0 | 105/61 | - | CS |
| N5 | 34 | 40+1 | 114/72 | - | CS |
| N6 | 39 | 39+3 | 113/73 | - | CS |
| N7 | 33 | 38+6 | 112/70 | - | CS |
| N8 | 29 | 38+5 | 106/82 | - | CS |
| N9 | 30 | 38+6 | 110/74 | - | CS |
| N10 | 30 | 38+4 | 99/58 | - | CS |
| N11 | 33 | 38+5 | 115/62 | - | CS |
| N12 | 31 | 39+1 | 115/60 | - | CS |
| N13 | 29 | 40+0 | 94/58 | - | CS |
| N14 | 34 | 39+2 | 115/65 | - | CS |
| N15 | 27 | 39+1 | 110/51 | - | CS |
| N16 | 38 | 39+1 | 112/63 | - | CS |
| PE1 | 39 | 36+6 | 150/110 | - | CS |
| PE2 | 34 | 39+1 | 169/111 | + | CS |
| PE3 | 32 | 38+6 | 140/81 | +++ | CS |
| PE4 | 31 | 38+3 | 150/90 | ++ | CS |
| PE5 | 33 | 38+2 | 166/101 | +++ | CS |
| PE6 | 36 | 33+3 | 171/105 | +- | CS |
| PE7 | 25 | 35+3 | 152/108 | +++ | CS |
| PE8 | 25 | 39+1 | 146/102 | ++++ | CS |
| PE9 | 37 | 36+6 | 135/79 | + | CS |
| PE10 | 25 | 35+1 | 151/98 | +++ | CS |
| PE11 | 32 | 34+3 | 156/100 | ++ | CS |
| PE12 | 25 | 39+2 | 145/92 | ++++ | CS |
| PE13 | 29 | 35+3 | 199/122 | +++ | CS |
| PE14 | 31 | 31+4 | 166/105 | +++ | CS |
| PE15 | 40 | 37+1 | 146/85 | + | CS |

Supplementary Table 2. Information of patients whose tissue sample were used for RT-PCR
